# Readiness of US Health Plans to Manage Cardiometabolic Risk

**Published:** 2009-06-15

**Authors:** Thomas E. Kottke, Courtney O. Jordan, Patrick J. O'Connor, Nicolaas P. Pronk, Rita Carreón

**Affiliations:** HealthPartners Research Foundation; University of Minnesota, Minneapolis, Minnesota; HealthPartners Research Foundation, Minneapolis, Minnesota; HealthPartners Research Foundation, Minneapolis, Minnesota, and Health Behavior Group, HealthPartners, Minneapolis, Minnesota; America’s Health Insurance Plans, Washington, DC

## Abstract

**Introduction:**

American health plans can make a substantial contribution to the control of cardiometabolic risk (CMR), a condition associated with both adverse health outcomes and increased cost of care. Our goal was to determine health plan interest in and ability to provide CMR control services.

**Methods:**

In January 2008, America's Health Insurance Plans, in collaboration with the HealthPartners Research Foundation, surveyed the chief medical officers of 74 member health insurance plans that offer commercial health maintenance organization, point of service, and preferred provider organization insurance. The response rate was 47%.

**Results:**

The 35 responding chief medical officers reported that their plans identify members with CMR through referral from case or care management (89%), health risk assessment data (86%), claims data (82%), and pharmaceutical use data (79%). Nearly all (97%) plans currently offer interventions for tobacco use, obesity/overweight, and nutrition. Ninety-four percent of plans offer interventions to increase physical activity. All plans offer health risk appraisal or assessment with feedback and education, 91% use Web-based tools, and 85% use health coaching to help plan members lower their risk. Perceived barriers to broader implementation of risk control programs included lack of resources (79%), limited available enrollee data (74%), and lack of reporting systems (79%). Few health plan officers viewed lack of purchaser interest to be a barrier to program implementation.

**Conclusion:**

Health plans appear to be positioned to provide CMR control services that could improve health outcomes, reduce health care costs, and increase workplace productivity in the United States.

## Introduction

Cardiometabolic risk (CMR) refers to a condition characterized by standard cardiovascular risk factors, such as blood pressure, cholesterol levels, and tobacco use, and metabolic risk factors, such as obesity, glucose intolerance, and insulin resistance. The path to CMR appears to start with the combination of inadequate physical activity and a diet that is low in fiber and high in saturated fat. Although CMR increases with body mass index (BMI), people with a normal BMI can develop it (1). Untreated, the combination of abdominal obesity and elevated triglycerides, low high-density lipoprotein levels, elevated blood pressure, and elevated plasma glucose often leads to coronary heart disease, stoke, premature illness and death, and higher health care costs (2,3). CMR is increasing among US adults, and this trend is driven largely by obesity, which is rapidly increasing in most population subgroups (4).

Physical, social, and economic environments can affect CMR because they promote or hinder participation in physical activity and adoption of healthy eating habits (5). Some experimental evidence demonstrates that increasing physical activity levels and improving nutrition often reduces CMR independent of weight loss (6-8). Health plans can support changes in nutrition, physical activity, and weight, which should be the primary focus of interventions that prevent and reduce CMR.

We surveyed medical leaders of health plans to assess whether they have the capability and motivation to provide health management services to people and communities to prevent and reduce CMR. The United States Preventive Services Task Force (USPSTF) concluded that evidence is insufficient to recommend for or against routine behavioral counseling to promote either physical activity or a healthy diet for unselected patients in primary care settings (9,10). However, an expert panel convened by the National Business Coalition on Health and the Centers for Disease Control and Prevention concluded that health risk assessment or appraisal (HRA) with feedback and health education shows "strong evidence of effectiveness in improving 1 or more health behaviors or conditions in populations of workers" (11). Although additional intervention components are beneficial, they are not necessary for efficacy.

In addition to efficacy, HRA has the advantage of flexibility because it does not need to be delivered in the doctor's office. When sponsored by employers and delivered either over the Internet or at the work site, HRA can reach groups — for example, young adult men — who visit a physician only when ill or injured. Health management services based on HRA with feedback and additional services can generate a favorable return on investment for employers (12-17).

Two critical components of any intervention program are the proportion of people in the population that the program reaches and the frequency at which the program engages participants. Because most Americans work or live with someone who does and because an even higher proportion have some type of health insurance, health plans, working with employers, have an opportunity to systematically reach a large proportion of the population with tailored health management services (18). We describe the types of services that health plans offer and discuss the capability of health plans to assume a leadership role in providing health management services to adults.

## Methods

The sample for this survey was drawn from health insurance plans in the United States that were members of America’s Health Insurance Plans (AHIP) as of November 15, 2007. Eligible health plans were identified from the AHIP member organization database. Eligibility requirements were 1) being an AHIP member health insurance plan that offered health maintenance organization (HMO), point-of-service (POS), or preferred provider organization (PPO) services and 2) having a combined enrollment of 50,000 members or more. Plans that did not provide commercial HMO, POS, or PPO health insurance products; leased network PPOs; subsidiary commercial plans; and plans with a combined commercial HMO, POS, or PPO enrollment of less than 50,000 were excluded. Leased network PPOs were excluded because they are typically used by self-funded employers who are seeking only discounted access to a provider network and are unlikely to provide health management or disease management services. Subsidiary commercial plans were excluded because employees of their parent companies were asked to answer on their behalf. Plans with fewer than 50,000 members were excluded because their disease and care management programs are often less developed than those of medium-sized and large health plans, and they are unlikely to have enough staff to respond to the industry surveys. These small plans make up only 0.9% of the aggregate HMO, POS, and PPO enrollment (19). According to calculations from tables in AIS’s Directory of Health Plans: 2007 (19), AHIP member health plans accounted for 90% of the total enrollment in the HMO/POS/PPO market.

The initial survey sample was 76 health insurance plans. In January 2008, the chief medical officers of each health plan were asked to either complete the survey or designate appropriate staff to the task. Two plans were excluded during the fielding of the survey because respondents indicated that their health plans did not meet the survey selection criteria. The final survey sample frame was 74 health insurance plans.

Medical officers from 35 health plans responded to the survey. Twenty health plans were HMOs, 8 were PPOs, and 2 were POS plans (Table). Two plans offered all 3 types of coverage and 3 plans offered 2 types of coverage. Total enrollment in the 35 health plans was approximately 47 million, and average enrollment was 1.3 million. The survey protocol was reviewed and approved by the institutional review board of the HealthPartners Research Foundation.

## Results

### Identifying CMR

Eighty percent of responding health plans reported that they identified members with elevated CMR. The methods used most frequently included referral from case or care management (89%), HRA data (86%), claims data (82%), and pharmacy use data (79%). However, medical officers of more than 70% of the plans also reported that they use provider referral to identify members with elevated CMR. Data from enrollment forms are rarely used.

### Intervention programs

More than 80% of medical officers of health plans reported that they address CMR among their enrollees as part of both a wellness/prevention domain and as part of a chronic care management domain. Nearly two-thirds of the plans reported that they also address CMR as part of a management or treatment domain.

**Figure 1:**
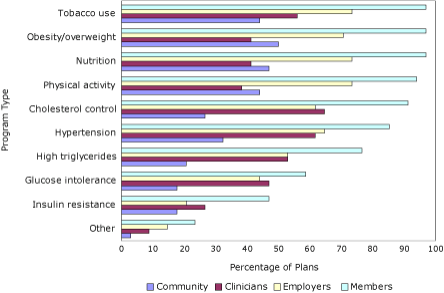
Percentage of health insurance plans with wellness, health promotion, or prevention programs/activities for members, employers, clinicians, and communities, by program type, 35 members of America's Health Insurance Plans, United States, January 2008.

Nearly all plans reported that they offer wellness, health promotion, or prevention programs to members for tobacco use, obesity/overweight, nutrition, and physical activity (Figure 1). Although fewer health plans offer these services to employers, the rank order of the frequency with which the services are offered is approximately the same. The pattern for programs offered to clinicians was different from the pattern offered to members. Programs most frequently offered to clinicians address cholesterol control, hypertension, tobacco use, and high triglycerides.

**Table:** 

**Program Type**	**Provide Program for the Community, %**	**Provide Program for Clinicians, %**	**Provide Program for Employers, %**	**Provide Program for Members, %**
Other	2.9	8.8	14.7	23.5
Insulin resistance	17.6	26.5	20.6	47.1
Glucose intolerance	17.6	47.1	44.1	58.8
High triglycerides	20.6	52.9	52.9	76.5
Hypertension	32.4	61.8	64.7	85.3
Cholesterol control	26.5	64.7	61.8	91.2
Physical activity	44.1	38.2	73.5	94.1
Nutrition	47.1	41.2	73.5	97.1
Obesity/overweight	50.0	41.2	70.6	97.1
Tobacco use	44.1	55.9	73.5	97.1

Nearly all of the plans address nutrition, cholesterol control, hypertension, tobacco use, physical activity, and obesity/overweight as part of their existing diabetes and cardiovascular disease chronic care programs. Approximately half of the plans address CMR as part of obesity programs. The proportions were lower for cancer, asthma, and chronic obstructive lung disease programs.

**Figure 2:**
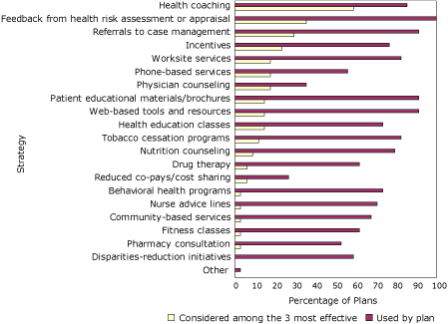
Percentage of health insurance plans that use strategies to help enrollees manage cardiometabolic risk and percentage of health insurance plans that rank the strategy as among the 3 most effective, by strategy, 35 members of America's Health Insurance Plans, United States, January 2008.

Medical officers of responding health plans reported using multiple strategies to assist enrollees in managing elevated CMR (Figure 2). All health plans use feedback from HRA, and nearly all plans provide Web-based tools and resources, patient educational materials/brochures, and referrals to case management/chronic care programs that their enrollees can access. Strategies most frequently named as among the 3 most effective in helping enrollees manage CMR were health coaching, feedback from HRA, referral to case management, and incentives. Although they were not perceived to be the most effective strategies, health plans also frequently reported using tobacco use cessation programs, work site services (eg, health education classes, cafeteria assessments, weight management programs), and nutrition counseling.

**Table:** 

### Clinician support

**Figure 3:**
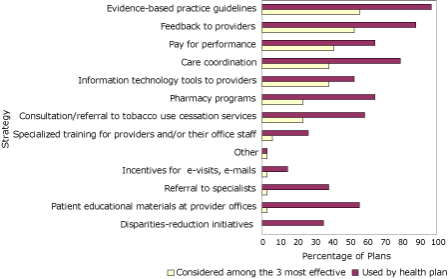
Percentage of health insurance plans that use strategies to assist clinicians evaluate and manage enrollees' cardiometabolic risk and percentage of health insurance plans that rank the strategy among the 3 most effective, by strategy, 35 members of America's Health Insurance Plans, United States, January 2008.

The 5 strategies that health plans reported using most frequently to assist clinicians to evaluate and manage CMR were evidence-based guidelines, feedback to providers (eg, physician reports, provider profiling), care coordination, pay for performance, and pharmacy programs (Figure 3). Most frequently reported among the 3 most effective strategies were the use of evidence-based guidelines, feedback to providers, and pay for performance programs. Although they were not perceived as the most effective strategies, health plans also frequently reported using the following strategies/interventions to assist clinicians to evaluate and manage CMR: consultation/referral to tobacco use cessation services, patient educational materials at provider offices, and information technology tools to providers.

**Table:** 

### Use of guidelines

More than 80% of medical officers of health plans cited the National Cholesterol Education Program Adult Treatment Panel III (NCEP-ATP III), USPSTF recommendation, and the Seventh Report of the Joint National Committee on Prevention, Detection, Evaluation, and Treatment of High Blood Pressure as guidelines that they use to identify, manage, or treat enrollees with elevated CMR. Approximately half of the health plan medical officers also reported using other guidelines. Other guidelines that were used included those developed by the National Institutes of Health, United States Public Health Service, American Heart Association, and American Diabetes Association, as well as guidelines developed by regional and local groups. Health plan medical officers reported infrequently using international, European, and subspecialty guidelines to identify, manage, or treat enrollees with CMR.

### Risk assessment tools

Approximately half of the health plans advocated a risk assessment tool to members or clinicians. When a risk calculator was advocated, it was approximately equally likely to be the NCEP tool or the Framingham risk calculator. Risk calculators that included CMR factors, for example Diabetes PHD (Personal Health Decisions), Reynolds Risk Score, and the PROCAM (Prospective Cardiovascular Münster) score, were recommended infrequently or not at all to members, clinicians, or employers. Approximately one-third of health plans recommended 1 or more of the risk assessment tools to address CMR.

### Barriers to implementation

**Figure 4:**
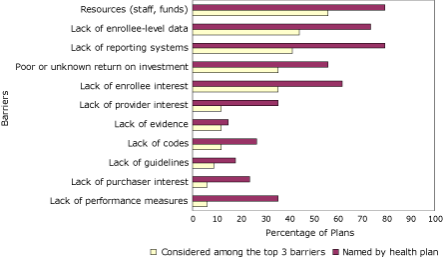
Percentage of health insurance plans that face barriers to addressing cardiometabolic risk and percentage of health insurance plans that rank the barrier among the top 3, by barrier, 35 members of America's Health Insurance Plans, United States, January 2008.

Medical officers of health plans most frequently reported shortage of resources, lack of reporting systems, and lack of enrollee-level data as barriers that they face in addressing CMR (Figure 4). These same 3 barriers were reported to be among the top 3 barriers to implementation. Although they were not identified as being the most important barriers, lack of patient adherence to medication regimen, lack of physician time to counsel and educate patients, lack of enrollee interest, and poor or unknown return on investment were frequently cited by health plans.

**Table:** 

### Plans for the future

Regarding plans to address CMR, medical officers of 57% of health plans reported that they are planning to expand their activities in the next 2 years, 29% reported being unsure of their plans, and the remaining 14% indicated that they have no plans to expand activities in this area.

## Discussion

Many factors limit the interpretation of these data. The survey response rate of 47% may have resulted from the possibility that responding medical officers were more likely than nonresponding medical officers to represent health plans that have active programs to manage CMR. Therefore, the data presented here may represent a best-case scenario, and the true activity levels of all plans may be substantially lower than those that may be inferred from the survey results. Furthermore, all data were self-reported, and no attempt was made to verify the responses from medical officers of the health plans. However, the medical officers who responded to the survey have the training and clinical insight to understand the concept of CMR and accurately respond to the survey questions. A search of PubMed failed to reveal results of similar surveys of health plans.

A notable finding is that health plans address traditional cardiovascular risk factors more aggressively than they do CMR. Therefore, much less attention is given to the CMR components of insulin resistance, glucose intolerance, and high triglycerides than it is to blood pressure control, control of low-density lipoprotein cholesterol, or interventions to increase physical activity and improve nutrition. Fortunately, by addressing poor nutrition and inadequate physical activity, health plans are addressing the root causes of CMR. People who are physically active and eat a diet high in fiber and low in saturated fat are less likely than those who are inactive and have a poor diet to develop high triglycerides, glucose intolerance, insulin resistance, obesity, or hypertension (20,21).

Moreover, most health plans can already administer HRA, and the fact that nearly all of the health plans use the Internet to communicate with their members is a positive sign from the perspective of program delivery. HRAs and the Internet create the capability to interact frequently with a large proportion of health plan members at relatively low cost. Although the effect is modest, interventions to increase physical activity and improve diet help people achieve these goals (22-27). The same is true for certain interventions designed to help people lose weight (28).

Our results suggest 2 principal strategies that health plans can adopt to help members reduce their CMR. First, the influence of insulin resistance, glucose intolerance, obesity, and high triglycerides in creating adverse cardiovascular outcomes should be emphasized to leaders of health plans. This task is primarily an informational and educational challenge for clinical and administrative leaders.

The second strategy is to develop effective communication strategies and tools to reach people with an intervention that is strong enough to change behavior. Many adults covered by a health insurance plan work or are living with an adult who works. Therefore, the scope of HRA and similar programs includes the employer in the intervention program. For a large proportion of Americans, the work site is their major locus of social interaction. Therefore, the broader involvement of employers and work sites could reinforce attempts by people to control their CMR. Health plans are well-positioned to encourage and educate physicians and medical groups to 1) more effectively deliver both lifestyle advice and support and 2) more systematically control risk factors such as high cholesterol and high blood pressure. These strategies would allow office-based physicians to focus on illness care and clinical preventive services while still having the opportunity to reinforce the crucial behavior change message.

Our results indicate that most health plans can deliver HRA with feedback and provide Internet and telephone-based intervention services. Because most Americans either have health insurance through their employer or live with someone who is employed, creating the conditions that encourage employers to purchase CMR health management services from health plans could have a substantial effect on health outcomes, health care costs, and workplace productivity in America.

## Figures and Tables

**Table.** Types of Coverage Offered by and Enrollments of Health Insurance Plans (N = 35), Survey on Cardiometabolic Risk-Control Services, United States, January 2008

**Type of Coverage**	No. of Health Plans (%)	No. of Enrolled Participants
HMO	20 (57.1)	20,034,695
PPO	8 (22.9)	24,008,055
POS	2 (5.7)	28,500
HMO, POS, and PPO	2 (5.7)	1,179,434
HMO and POS	2 (5.7)	385,000
HMO and PPO	1 (2.9)	1,566,154

Abbreviations: HMO, health maintenance organization; PPO, preferred provider organization; POS, point-of-service plan
